# Supplementary figures and images for: A quality improvement study on the relationship between intranasal povidone-iodine and anesthesia and the nasal microbiota of surgery patients
Source: PLoS One. 2022 Dec 9;17(12):e0278699. doi: 10.1371/journal.pone.0278699 (PMC9733847; doi:10.1371/journal.pone.0278699)

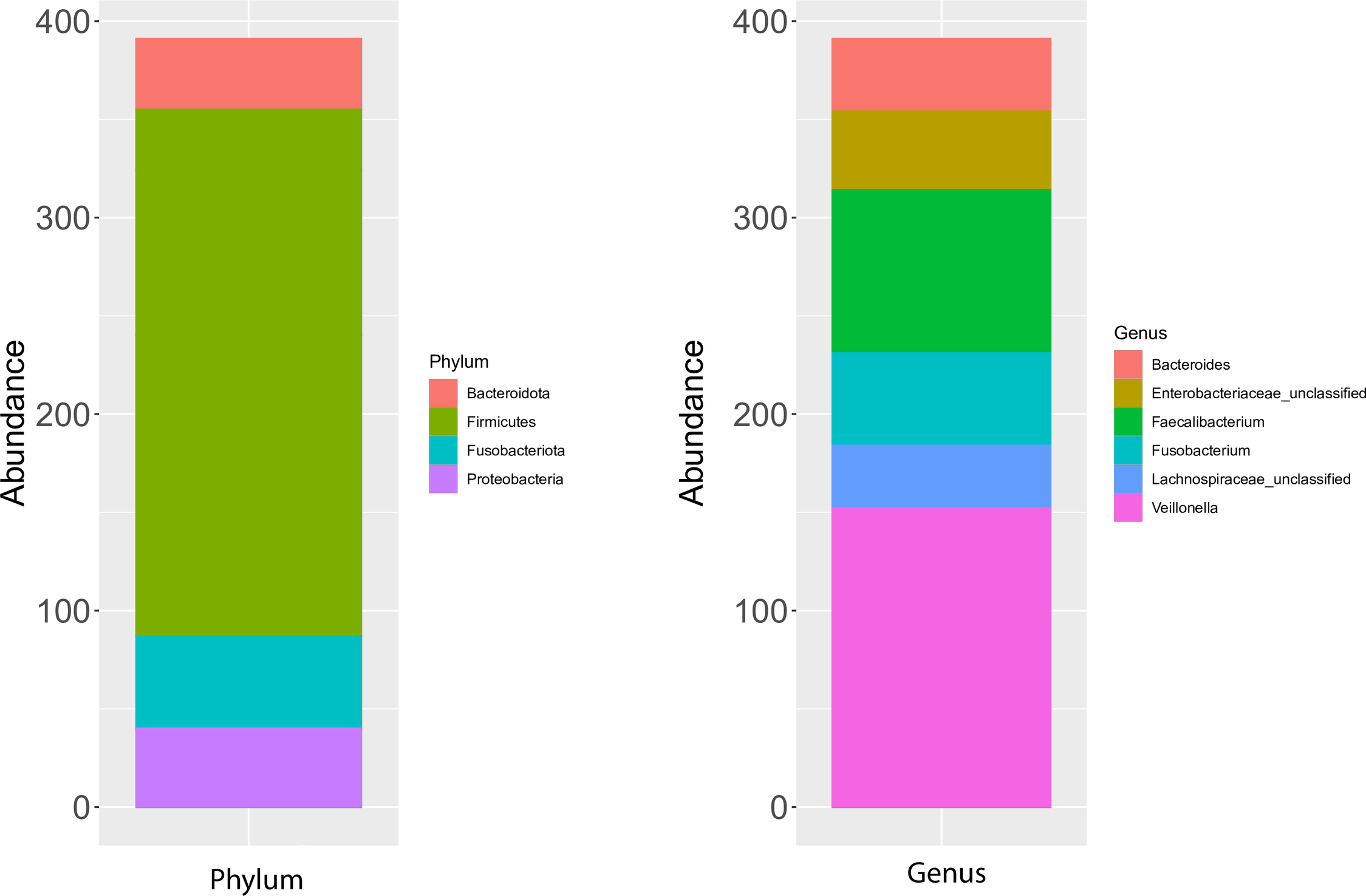

Supplement: S1 Fig — Stacked bar plots of the absolute abundance of top 10 phyla and genera composition in positive control (ZymoBIOMICS® Gut Microbiome). Rare taxa are classified as “uncultured”. The legend displays the color coding of genera and phyla to which these taxa belong. (TIF) [file pone.0278699.s001.tif]
